# Effects of *Saccharomyces cerevisiae* on alleviating cytotoxicity of porcine jejunal epithelia cells induced by deoxynivalenol

**DOI:** 10.1186/s13568-019-0863-9

**Published:** 2019-09-03

**Authors:** Yang Liu, Juan Chang, Ping Wang, Qing-qiang Yin, Wei-wei Huang, Chao-qi Liu, Xian-xiao Bai, Qun Zhu, Tian-zeng Gao, Pu Zhou

**Affiliations:** 1 College of Animal Science and Veterinary Medicine, Henan Agricultural University, Zhengzhou, 40002 China; 2 Institute of Animal Husbandry and Veterinary Science, Henan Academy of Agricultural Sciences, Zhengzhou, 450002 China; 3 Henan Delin Biological Product Co. Ltd., Xinxiang, 453000 China; 4 Henan Guangan Biotechnology Co., Ltd., Zhengzhou, 450001 China; 5 Wugang Animal Product Quality Inspection Center, Wugang, 467000 China

**Keywords:** Deoxynivalenol, Detoxification, *Saccharomyces cerevisiae*, IPEC-J2, Cytotoxicity

## Abstract

Deoxynivalenol (DON) is one of the mycotoxins most frequently encountering in cereal-based foods throughout the world. *Saccharomyces cerevisiae* was used to alleviate porcine jejunal epithelia cell (IPEC-J2) injury induced by DON in this study. The results indicated that cell viability and proliferation rates were significantly decreased when DON concentrations were increased from 0 to 64 µM after 24 h incubation (*p *< 0.05). The longer incubation time and higher DON concentrations would cause more serious effects on cell viability. *S. cerevisiae* could significantly degrade DON and decrease lactic dehydrogenase (LDH) release in the cells induced by DON (*p *< 0.05). DON (4 µM) could increase necrotic and apoptotic cell rates as well as decrease viable cell rates, compared with the control group (*p *< 0.05). However, *S. cerevisiae* addition in the DON group could decrease necrotic, late apoptotic and early apoptotic cell rates by 38.05%, 46.37% and 44.78% respectively, increase viable cell rates by 2.35%, compared with the single DON group (*p *< 0.05). In addition, *S. cerevisiae* addition could up-regulate mRNA abundances of IL-6, IL-8 and IL-10 in IPEC-J2 cells (*p *< 0.05), but down-regulate mRNA abundances of tight junction proteins (TJP-1) and occludin by 36.13% and 50.18% at 1 µM of DON (*p* < 0.05). It could be concluded that *S. cerevisiae* was able to alleviate IPEC-J2 cell damage exposed to DON.

## Introduction

Deoxynivalenol (DON), also known as vomitoxin, is a mycotoxin produced by *Fusarium culmorum*, *Fusarium graminearum*, *Fusarium crookwellense* and *Fusarium sambucinum*, and so on. DON is a widespread feed contaminant and considered as an important risk factor for both human and animal health. When the animals are exposed to feed or food contaminated with DON, it may cause anorexia, alter immunologic function, reduce weight gain and nutrient efficiency. DON can bind intracellular ribosomes to inhibit protein biosynthesis and induce pro-inflammatory cytokine production (Pestka 2007; Pestka and Shephard 2010). Pigs are the most sensitive animals to DON among the different kinds of animals, they are considered as the most relevant animal model of human sensitivity (Lucioli et al. 2013; Nossol et al. 2013). It has been reported that chronic exposure to DON at 1–2 mg/kg diet will result in low appetite, 3 mg/kg DON will reduce body temperature and alter gastric wall, while long-term DON exposure at 4 mg/kg diet will decrease feed intake, weight gain, and feed efficiency for pigs (Bergsjø et al. 1992).

The gastrointestinal tract represents the first barrier against contaminated food and feed (Odenwald and Turner 2013). Several studies have described the direct effects of DON-contaminated food or feed on gastrointestinal tract and intestinal epithelial cells (Prelusky et al. 1988; Pinton et al. 2012). The previous reports showed that proliferation and viability of intestinal cells were impaired by DON (Diesing et al. 2011a; Pinton et al. 2012, 2015). DON is also directly involved in intestinal inflammation (Graziani et al. 2015). It has been reported that DON potentiates the inflammatory response to *Salmonella typhimurium* in the porcine intestinal ileal loop model by up-regulating the expression of IL-1β, IL-12, IL-8, MCP-1, TNFα and IL-6 (Vandenbroucke et al. 2011).

Probiotics are being widely used as an alternative to promote health and performance of farm animals worldwide. Some researchers have demonstrated that probiotics can positively balance gastrointestinal microbiota, and thereby improve animal production and health (Chaucheyras-Durand and Durand 2010). Yeasts have been widely used to promote gut health both in humans and animals (Czerucka et al. 2007; Chaucheyras-Durand and Durand 2010). It has been reported that there is a beneficiary effect of live yeast supplementation on the health and performance of pigs (Li et al. 2006). Positive effects of yeast supplementation may be partly due to the ability of yeast to modify the composition of gut microbiota and enhance the immune responsiveness of the piglets (Trevisi et al. 2015). *Saccharomyces cerevisiae* has been reported to have positive effect on health and performance of ruminants and pigs by modifying hindgut microbiota (Kiros et al. 2018).

Due to food and feed contaminated with DON posing a health risk to humans and livestock, how to alleviate DON hazard becomes more and more important. A lot of researches have shown that biological detoxifications of DON and other mycotoxins by *Nocardioides* sp. WSN05-2, *S. cerevisiae* and compound probiotics are more effective than other methods (Alassane-Kpembi et al. 2018; Huang et al. 2018). Therefore, selecting the optimal beneficial microorganisms to alleviate mycotoxin harm and studying their detoxifying mechanisms become more and more important.

In order to reduce DON cytotoxicity, *S. cerevisiae* was selected in this research to study its effect on alleviating cell apoptosis and necrosis induced by DON through positively regulating mRNA abundances of some cytokines and tight junction proteins (TJP) in porcine jejunal epithelial cells (IPEC-J2).

## Materials and methods

### Cell culture

The porcine jejunal epithelial cells (IPEC-J2) were purchased from Shanghai Guandao Biological Engineering Co., Ltd. (Shanghai, China). They were cultured in 15 mM Dulbecco’s Modified Eagle Medium/Nutrient Mixture F-12 (DMEM/F12 at 1/1) (Hyclone, Logan, UT, USA), supplemented with 10% (v/v) fetal bovine serum (FBS, Zhejiang Tianhang Biotechnology Co., Ltd, Hangzhou, China) and 1% (v/v) penicillin/streptomycin (Hyclone, Logan, UT, USA), and incubated at an atmosphere of 5% CO_2_ and 37 °C. The cells were seeded at a density of 0.7 × 10^5^/cm^2^ in 25 cm^2^ plastic tissue culture flasks (Corning Costar Corp., Cambridge, MA, USA). Cell culture medium was changed 3 times weekly. The cells were prepared by trypsinization with 0.25% trypsin–EDTA (Solarbio^®^ Life Sciences, Beijing, China) for 3–5 min at 37 °C, and then they were seeded at a suitable concentration according to the following experiments.

### Preparation of DON

Purified DON (D0156; Sigma-Aldrich, Darmstadt, Germany) was diluted in absolute ethanol to prepare stock solution at 16.8 mM, stored at − 20 °C. Serial dilutions of DON at concentrations of 0.25, 0.5, 1, 2, 4, 8, 16, 32, 64 µM were prepared in serum-free cell medium (ethanol concentration < 1% in all experiments). DON was determined by ELISA kit (R-Biopharm AG, Darmstadt, Germany).

### *Saccharomyces cerevisiae* culture

*Saccharomyces cerevisiae* was purchased from China General Microbiological Culture Collection Center (CGMCC 2.1542), incubated in yeast extract-peptone dextrose (YPD) broth at 30 °C with slightly shaking. It was harvested in log phase, determined by plating serial dilutions and measured as colony forming units (CFU). Prior to use, *S. cerevisiae* was centrifuged at 5000×*g* for 3 min at 4 °C, washed twice to remove excessive YPD, and re-suspended in DMEM/F-12 medium with 15 mM 2-[4-(2-hydroxyethyl)-1-piperazinyl]ethanesulfonic acid (HEPES).

### Cell viability affected by DON

IPEC-J2 cells were incubated and seeded in 96-well plate at a density of 1 × 10^4^/well. Cell culture medium was removed, and the cells were washed twice with phosphate-buffered saline (PBS). The cells were exposed to fresh DMEM/F-12 media without serum and antibiotics containing DON at concentrations from 0.25 to 64 µM for 24 h, followed by adding 10 μL MTT (3-(4,5-dimethyl-2-thiazolyl)-2,5-diphenyl-2*H*-tetrazolium bromide, 5 mg/mL PBS) (Solarbio^®^ Life Sciences, Beijing, China), and then additionally incubated for 4 h. The cell culture was removed carefully with pipette, and 150 µL dimethyl sulfoxide (DMSO, Shanghai Solarbio Biotechnology Co., Ltd. Shanghai, China) were added to each well to dissolve the crystalline formazan, and then the plate was shaken for 10 min. Cell viability was measured by MTT assay and indicated by optical density (OD) value at 450 nm using the microplate ELISA reader (Multiscan MS, Thermo Labsystems, Helsinki, Finland). The cell proliferation rate was calculated using the following formula: Cell proliferation rate (%) = a/b × 100, a  =  OD_450_ derived from the wells with cells and DON, b  =  OD_450_ derived from control wells with cells. Six wells were prepared for each treatment.

In order to determine the effect of different doses and reaction time of DON on cell viability, the cells were exposed to DMEM/F-12 media without serum and antibiotics containing DON at concentrations of 0, 0.25, 0.5, 1, 2 and 4 µM. The reaction time was 2, 4, 8, 16 and 32 h, respectively. The cell viability was measured by MTT assay according the above protocol.

### Effect of *S. cerevisiae* on LDH release in cells induced by DON

The pre-washed IPEC-J2 cells at a density of 1 × 10^5^/well were exposed to the following 4 kinds of DMEM/F-12 media without serum and antibiotics: (1) only media used as the control group; (2) containing DON at concentrations of 0, 0.25, 0.5, 1, 2 and 4 µM; (3) containing 1 × 10^5^ CFU/mL *S. cerevisiae*; (4) containing DON at concentrations from 0 to 4 µM in combination with 1 × 10^5^ CFU/mL *S. cerevisiae*. The reaction time was 8 h.

Cellular membrane integrity was assessed by measuring lactic dehydrogenase (LDH) activity in cell culture medium. LDH assay was performed according to manufacturer’s protocol (Beyotime Biotechnology, Jiangsu, China). At the end of the experiment, 120 μL media from each well was transferred into a new 96-well plate, and OD values were recorded at 490 nm by the plate reader (Multiscan MS, Thermo Labsystems, Helsinki, Finland) for measuring LDH activity. DON residual amount in the media was measured according to the above protocol.

### Annexin V/PI staining and flow cytometric analysis

IPEC-J2 cells were treated with 4 µM DON, *S. cerevisiae* or *S. cerevisiae* with DON for 8 h. The cells were washed three times with PBS and re-suspended in 500 μL binding buffer, stained with 5 µL propidium iodide (PI) and 5 μL Annexin V-fluorescein isothiocyanate (FITC) according to the annexin V-FITC/PI staining kit manufacturer’s instruction (KeyGEN Bio TECH, Nanjing, China), and analyzed by FACSCanto II cytometer (BD Biosciences, San Jose, USA) to identify cell statuses. The data were analyzed by BD FACSuite software. Cell statuses were classified as necrotic (FITC^+^/PI^+^), early apoptotic (FITC^+^/PI^−^), late apoptotic, and viable (FITC^−^/PI^−^) cells.

### Determination of cytokine and TJP mRNA abundances in IPEC-J2 cells

IPEC-J2 cells (1 × 10^5^ cells/mL) were cultivated with *S. cerevisiae* (1 × 10^5^ CFU/mL) at a ratio of 1:1 in the cell culture media containing 0, 0.25, 0.5, 1, 2, 4 µM DON for 8 h, and then washed three times with PBS to investigate the effect of *S. cerevisiae* on mRNA abundances of cytokines such as interleukin-6 (IL-6), IL-10 and IL-8, tight junction protein (TJP-1 and occludin). The total RNA of each simple was isolated by using Trizol reagent (Takara Biotechnology Co., Ltd. Dalian, China). The quantified RNA (1 µg) was used to synthesize cDNA by Thermal Cycler (BIO-RAD, California, USA). For qPCR reaction, the mixture (10 μL) was prepared as follows: 0.5 μL cDNA and RT enzyme, 5 μL SYBR^®^ Rremix Ex TaqTM (2×), 0.5 μL forward and reverse primers (10 μM) and 3.5 μL RNase-free water. The primers were shown in Table 1. The reaction cycles were performed first at 50 °C for 5 min, followed by 95 °C for 5 min, then 40 cycles at 95 °C for 15 s, 60 °C for 30 s and 72 °C for 30 s. The glyceraldehyde-3-phosphate dehydrogenase (GAPDH) gene was used as the housekeeping gene (Bustin et al. 2009). The relative expression ratio was calculated with the 2^−∆∆CT^ method (Schmittgen and Livak 2008).

**Table 1:** Primer sequences of some genes for RT-PCR

Genes	Accession number	Primer sequences	Sizes (bp)
TJP-1	XM_021098896.1	F: CATAAGGAGGTCGAACGAGGCATC	181
		R: CTGGCTGAGCTGACAAGTCTTCC	
IL-8	NM_213867.1	F: GACCCCAAGGAAAAGTGGGT	186
		R: TGACCAGCACAGGAATGAGG	
IL-6	NM_214399.1	F: TGCAGTCACAGAACGAGTGG	116
		R: CAGGTGCCCCAGCTACATTAT	
IL-10	NM_214041.1	F: GCCAAGCCTTGTCAGAGATGATCC	198
		R: AGGCACTCTTCACCTCCTCCAC	
Occludin	NM_001163647.2	F: CAGCCTCATTACAGCAGCAGTGG	158
		R: ATCCAGTCTTCCTCCAGCTCGTC	
GAPDH	XM-004387206.1	F: ATGGTGAAGGTCGGAGTGAA	154
		R: CGTGGGTGGAATCATACTGG	

### Statistical analysis

Each average value was from 3 independent experiments, 6 replicates for each experiment. All data were presented as mean ± standard deviation (SD), and analyzed by IBM SPSS Statistic Version 20.0 Statistical Software (Sishu software Co., Ltd. Shanghai, China). The data were submitted to one-way analysis of variance (ANOVA), followed by Tukey’s test. *p* < 0.05 was considered as significant difference.

## Results

### Effect of DON and *S. cerevisiae* on IPEC-J2 cell viability and proliferation rates

**Fig. 1:**
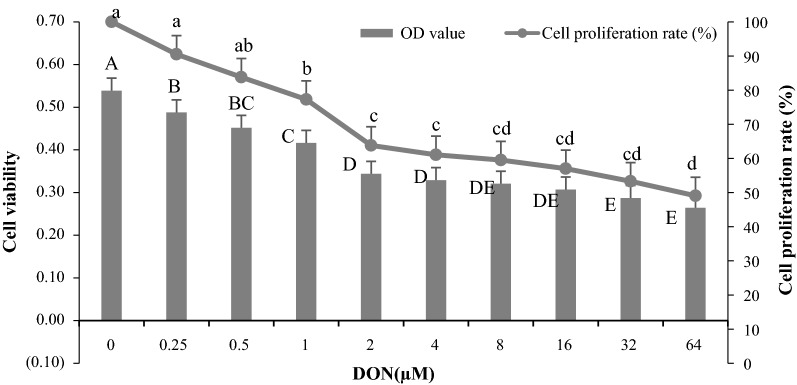
Effect of different DON concentrations on IPEC-J2 cell viability and proliferation rates after 24 h incubation. The different capital letters indicated the significant differences for cell viability among the different groups (*p* < 0.05), while the same capital letters indicated the insignificant differences for cell viability among the different groups (*p* > 0.05). The different lowercase letters indicated the significant differences for cell proliferation rates among the different groups (*p* < 0.05), while the same lowercase letters indicated the insignificant differences for cell proliferation rates among the different groups (*p *> 0.05)

Figure 1 indicated that cell viability and proliferation rates were significantly decreased when DON concentrations were increased from 0 to 64 µM after 24 h incubation (*p* < 0.05). Compared with the control group (0 µM), cell viability was significantly affected by different levels of DON. However, cell proliferation rates were insignificantly affected when DON levels were lower than 0.5 µM (*p *> 0.05), but they were 22.74%, 36.18%, 38.96%, 40.46%, 43.00%, 46.70% and 50.94% significantly lower than the control group (*p *< 0.05) when DON levels were increased from 1 to 64 µM.

Table 2 revealed that the cell viability was not affected by any concentrations of DON within 2 h incubation (*p* > 0.05); however, it was significantly decreased especially when DON concentrations were increased or incubation time was prolonged (*p *< 0.05). It was deduced that the longer incubation time and higher DON concentrations would cause more serious effects on cell viability.

**Table 2:** Effect of different DON concentrations and incubation time on IPEC-J2 cell viability

DON levels (µM)	2 h	4 h	8 h	16 h	32 h
0	0.62 ± 0.03^Ab^	0.80 ± 0.06^Aa^	0.87 ± 0.03^Aa^	0.84 ± 0.03^Aa^	0.67 ± 0.02^Ab^
0.25	0.68 ± 0.05^Abc^	0.71 ± 0.04^ABb^	0.83 ± 0.03^ABa^	0.72 ± 0.06^Bb^	0.63 ± 0.01^Bc^
0. 5	0.67 ± 0.08^Ac^	0.70 ± 0.02^ABb^	0.76 ± 0.02^BCa^	0.76 ± 0.04^ABa^	0.59 ± 0.02^Bc^
1	0.70 ± 0.04^Aab^	0.68 ± 0.06^Bb^	0.70 ± 0.03^CDab^	0.63 ± 0.05^Cb^	0.54 ± 0.01^Cc^
2	0.72 ± 0.04^Aa^	0.68 ± 0.05^Ba^	0.70 ± 0.05^CDa^	0.57 ± 0.04^Cb^	0.48 ± 0.02^Dc^
4	0.63 ± 0.04^Abc^	0.69 ± 0.01^Ba^	0.67 ± 0.07^Dab^	0.58 ± 0.03^Cc^	0.43 ± 0.02^Ed^

The different capital letters in the same columns indicated the significant differences for cell viability at the different DON concentrations (*p* < 0.05), while the same capital letters in the same columns indicated the insignificant differences for cell viability at the different DON concentrations (*p *> 0.05). The different lowercase letters in the same rows indicated the significant differences for cell viability at the different incubation time (*p *< 0.05), while the same lowercase letters in the same rows indicated the insignificant differences for cell viability at the different incubation time (*p* > 0.05)

### LDH release from IPEC-J2 cells exposed to DON and *S. cerevisiae*

Table 3 showed that LDH release was almost the same among the control and 0.25 µM DON groups whether *S. cerevisiae* was added or not (*p* > 0.05). However, LDH release was respectively decreased by 66.67%, 69.86%, 75.95% and 68.00% in the groups containing *S. cerevisiae* and DON (*p *< 0.05), compared with their own corresponding groups containing DON concentrations at 0.5, 1, 2 and 4 µM, indicating that *S. cerevisiae* was able to alleviate DON negative effect on cell damage.

**Table 3:** Effect of *S. cerevisiae* on decreasing LDH release of cells induced by DON for 8 h

Items	LDH release (OD value)
Control	0.28 ± 0.04^BC^
DON (0.25 µM)	0.30 ± 0.01^B^
DON (0. 5 µM)	0.69 ± 0.08^A^
DON (1 µM)	0.73 ± 0.06^A^
DON (2 µM)	0.79 ± 0.04^A^
DON (4 µM)	0.75 ± 0.01^A^
*S. cerevisiae*	0.24 ± 0.02^BC^
*S. cerevisiae *+ DON (0.25 µM)	0.22 ± 0.01^BC^
*S. cerevisiae *+ DON (0. 5 µM)	0.23 ± 0.02^BC^
*S. cerevisiae *+ DON (1 µM)	0.22 ± 0.01^BC^
*S. cerevisiae *+ DON (2 µM)	0.19 ± 0.02^C^
*S. cerevisiae *+ DON (4 µM)	0.24 ± 0.00^BC^

The different capital letters in the column indicated the significant differences (*p* < 0.05), while the same capital letters in the column indicated the insignificant differences (*p *> 0.05)

### DON degradation by *S. cerevisiae* during incubating with IPEC-J2 cells

DON concentrations in *S. cerevisiae* + DON groups (0.25, 0.5 and 1 µM) were decreased by 60.87%, 44.19% and 43.02%, compared to the corresponding single DON groups (*p *< 0.05), respectively. When DON concentrations were 2 and 4 µM, they were deceased by 25.50% and 10.27% with *S. cerevisiae* addition (*p *> 0.05), indicating that *S. cerevisiae* was more effective for degrading DON especially when DON concentration was less than 1 µM (Table 4).

**Table 4:** DON degradation by *S. cerevisiae* when incubating with IPEC-J2 cells for 8 h

Groups	DON residue (µM)	Groups	DON residue (µM)
Control	Undetected	*S. cerevisiae*	Undetected
DON (0.25 µM)	0.23 ± 0.05^a^	*S. cerevisiae *+ DON (0.25 µM)	0.09 ± 0.01^b^
DON (0.5 µM)	0.43 ± 0.02^a^	*S. cerevisiae *+ DON (0.5 µM)	0.24 ± 0.01^b^
DON (1 µM)	0.86 ± 0.05^a^	*S. cerevisiae *+ DON (1 µM)	0.49 ± 0.04^b^
DON (2 µM)	1.49 ± 0.27^a^	*S. cerevisiae *+ DON (2 µM)	1.11 ± 0.20^a^
DON (4 µM)	3.70 ± 0.37^a^	*S. cerevisiae *+ DON (4 µM)	3.32 ± 0.78^a^

The different lowercase letters in the same rows indicated the significant differences (*p* < 0.05), while the same lowercase letters in the same rows indicated the insignificant differences (*p* > 0.05)

### Effect of *S. cerevisiae* addition on alleviating cell damage induced by DON

**Fig. 2:**
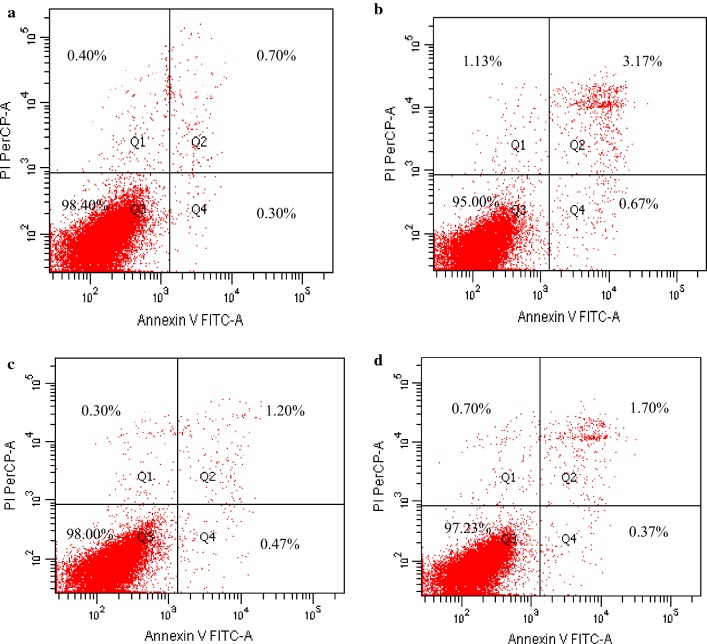
Effect of *S. cerevisiae* on protecting IPEC-J2 cell from damage induced by DON after incubating for 8 h. IPEC-J2 cells were treated without DON and *S. cerevisiae* (**a**, the control group), 4 µM DON (**b**), *S. cerevisiae* (**c**), and *S. cerevisiae *+ 4 µM DON (**d**), respectively. The data presented in this figure are the means of percentages in different cell statuses, 6 replicates for each treatment. Q1-4 represents necrotic, late apoptotic, viable and early apoptotic cells, respectively

Figure 2 indicated that DON (4 µM) could increase necrotic, late apoptotic and early apoptotic cell rates by 182.50%, 352.86% and 123.33%, while decrease viable cell rates by 3.46%, compared with the control group (*p *< 0.05). However, *S. cerevisiae* addition in DON group could decrease necrotic, late apoptotic and early apoptotic cell rates by 38.05%, 46.37% and 44.78%, while increase viable cell rates by 2.35%, compared with single DON group (*p *< 0.05), respectively. It was indicated that *S. cerevisiae* could protect cells from DON damage.

### Cytokine and TJP mRNA abundances of IPEC-J2 cells exposed to DON and *S. cerevisiae*

**Fig. 3:**
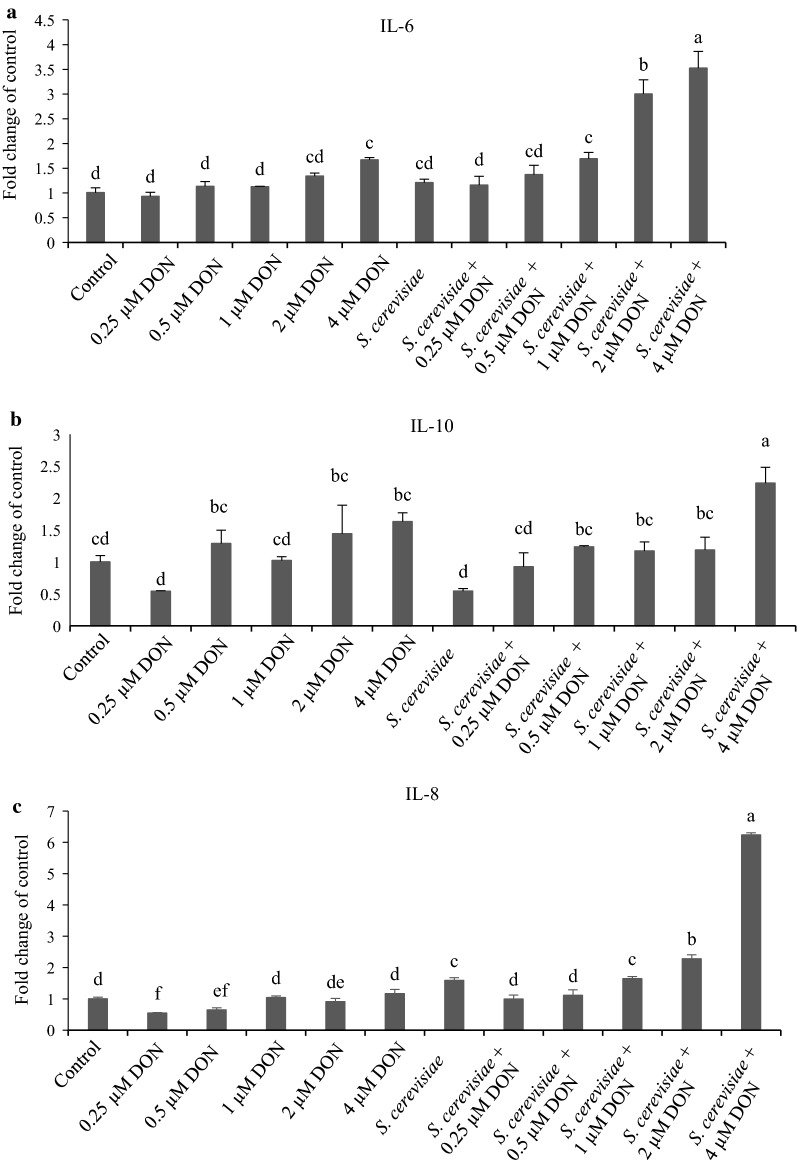

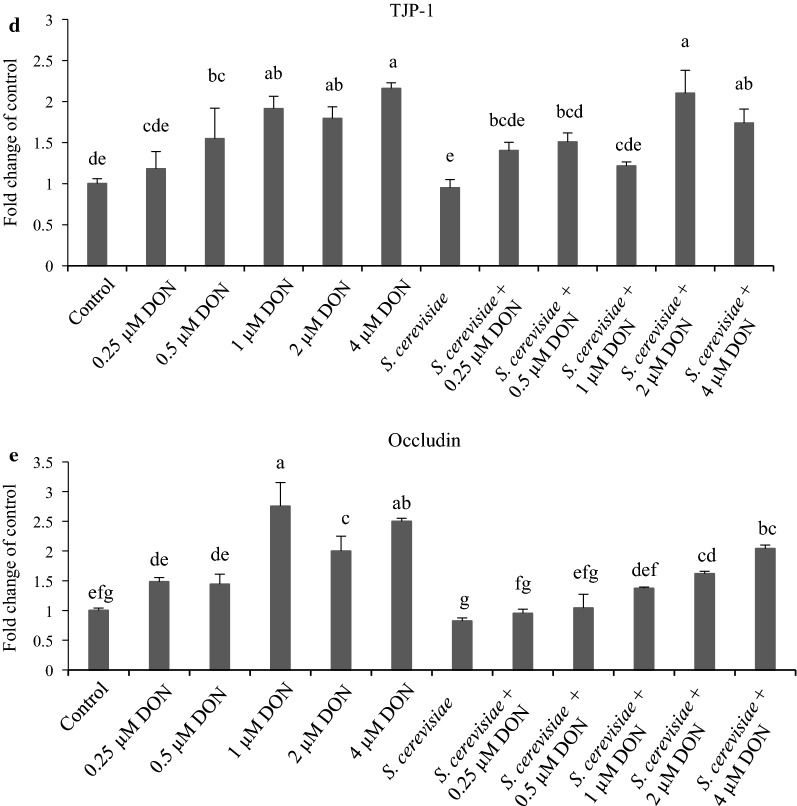
Cytokine and TJP mRNA abundances of IPEC-J2 cells affected by DON and *S. cerevisiae* after incubating for 8 h. The different lowercase letters among all the bars indicated the significant differences (*p* < 0.05), while the same lowercase letters among all the bars indicated the insignificant differences (*p* > 0.05). **a** IL-6, **b** IL-10, **c** IL-8, **d** TJP-1, **e** occludin

Figure 3a showed that mRNA abundances of IL-6 in 0.6 and 4 µM DON groups were higher than that in the control group (*p *< 0.05), which was further up-regulated by *S. cerevisiae* addition in the groups with 1, 2 and 4 µM DON, compared with the corresponding single DON groups (*p *< 0.05). Figure 3b indicated that mRNA abundances of IL-10 in all the DON groups were almost the same as the control group (*p *< 0.05), which was significantly up-regulated by *S. cerevisiae* addition in 4 µM DON group, compared with the other groups (*p *< 0.05). Figure 3c showed that mRNA abundances of IL-8 in 0.25 and 0.5 µM DON groups were lower than that in the control group (*p *< 0.05); however, they were up-regulated in all the groups containing *S. cerevisiae*, compared with the corresponding single DON groups (*p *< 0.05). Figure 3d showed that mRNA abundances of TJP-1 in 0.5, 1, 2 and 4 µM DON groups were higher than that in the control group (*p* < 0.05); however, they were 36.13% down-regulated by *S. cerevisiae* addition only in the 1 µM DON group, compared with its corresponding single DON group (*p *< 0.05). Figure 3e indicated that mRNA abundances of occludin in 1, 2 and 4 µM DON groups were higher than that in the control, 0.25 and 0.5 µM DON group (*p *< 0.05); however, they were 35.81% and 50.18% down-regulated by *S. cerevisiae* addition in the groups with 0.25 and 1 µM DON, compared with their corresponding single DON groups (*p *< 0.05). In a preliminary summary, *S. cerevisiae* addition could up-regulate mRNA abundances of IL-6, IL-8 and IL-10 of IPEC-J2 cells, but down-regulate mRNA abundances of TJP-1 and occludin at 1 µM of DON.

## Discussion

Deoxynivalenol is known to affect animal production performance as well as the proliferation and viability of animal intestinal epithelial cells. Therefore, DON biodegradations by microorganisms have been conducted for many years (Styriak et al. 2001; Weaver et al. 2014; Alassane-Kpembi et al. 2018). This research revealed that there was a DON dose-dependent pattern for *S. cerevisiae* to degrade DON, i.e. DON degradation rates were decreased from 60.87 to 10.27% when DON concentrations were increased from 0.25 to 4 µM. The reason may be due to high DON concentrations surpassing the ability of *S. cerevisiae* to degrade DON. This finding will help to partly explain the reason why *S. cerevisiae* is able to protect intestinal epithelial cells from damage induced by DON.

Intestinal epithelial cells are the first physical barrier against external stimuli, which may activate innate defense mechanisms to reduce the risk of the undesirable passage of xenobiotics, mycotoxins, harmful molecules and pathogenic microorganisms across intestinal membrane. A lot of researches have confirmed that the negative effects of DON on intestinal epithelial cells include modulating cell proliferation and viability, impairing intestinal barrier functions (Diesing et al. 2011b; Pinton et al. 2012, 2015), inhibiting nutrient absorption (Awad et al. 2014), and taking part in intestinal inflammation (Graziani et al. 2015). Therefore, how to alleviate DON cytotoxicity will be important for protecting intestinal cells from DON damage.

Cell death occurs mainly through two major modes including necrosis and apoptosis. Cell apoptosis also called as “programmed cell death”, means that the cell death occurs during embryogenesis, metamorphosis, endocrine-dependent tissue atrophy and normal tissue turnover (Nagata 1997). DON-induced cell apoptosis and necrosis have already been demonstrated in colon carcinoma cells or intestinal epithelial cell lines, which may seriously inhibit immune function (Diesing et al. 2011b; Bensassi et al. 2012; Broekaert et al. 2016; Zhang et al. 2016). The previous result showed that pretreatment with 8 μM DON could decrease the viability of porcine alveolar macrophage cells and increase cell apoptosis, but *Saccharomyces boulardii* could rescue apoptotic cells induced by DON (Chang et al. 2017). The further research showed that *S. boulardii* could suppress DON-induced p38 mitogen-activated protein kinase pathway activation, reduce the expression of downstream inflammatory cytokines, and promote the expression of anti-apoptotic genes to inhibit apoptosis induced by DON in porcine alveolar macrophage cells (Chang et al. 2017), which corresponds to this research even though the different cell types and microbial species are used.

LDH is an oxidoreductase enzyme that catalyses the interconversion of pyruvate and lactate, which will be released from cells to enter the bloodstream after cell damage or red blood cell hemolysis. Since LDH is a fairly stable enzyme, it has been widely used to evaluate the presence of damage and toxicity of tissues and cells (Diesing et al. 2011b; Zanello et al. 2011). Therefore, the release amount of LDH can indirectly reflect the degree of cell membrane damage. It was reported that 2 to 4 μg/mL DON could significantly increase LDH release and decrease cell number and proliferation after reaction for 24–72 h (Diesing et al. 2011a), in agreement with this study. Another report showed that 50% reduction of MTT signal was found at 0.66 µg/mL DON after 24 h incubation with Caco-2 cells (Sergent et al. 2006); however, IPEC-J2 cell proliferation was decreased by 50.94% at 64 µM DON in this study, which may be due to the different effects from the different cell types.

The production of cytokines or chemokines is one of the major innate immune responses against microorganisms, mycotoxins and other harmful factors in epithelial cells. IL-6, IL-10 and IL-8 responses in intestinal epithelial cells play important roles in the pathogenesis and immune defense against stimuli. IL-6 is a pleiotropic cytokine that is commonly produced at local tissue sites and released into circulation in almost all situations of homeostatic perturbation typically including endotoxemia, endotoxic lung, trauma and acute infections (Kishimoto et al. 1992). IL-8 is a multifunctional cellular chemotactic factor, which plays major roles in chemotaxis and activation of neutrophils, lymphocytes and other immune cells, as well as in pathogen defense, inflammatory response and immune regulation (Sallusto and Baggiolini, 2008). It was found that *Lactobacillus acidophilus* and *Lactobacillus rhamnosus* had different influence on the innate cytokine IL-6 and IL-8 production in rotavirus (PRV) OSU-infected IPEC-J2 cells (Liu et al. 2010). *L. acidophilus* increased the IL-6 response to rotavirus infection, which is consistent with the immunostimulatory effect of *L. acidophilus* on B- and T-cell immune responses (Zhang et al. 2008); while *L. rhamnosus* significantly decreased IL-6 production by the IPEC-J2 cells. It was further proved that treatment of normal IPEC-J2 cells with *L. rhamnosus* enhanced the IL-6 and IL-8 productions, whereas *L. rhamnosus* treatment of rotavirus-infected cells reduced the IL-6 and IL-8 responses to rotavirus, which supports the findings that *L. rhamnosus* has the different regulatory and stimulatory effects in hosts with different immune statuses (Pelto et al. 1998).

*Saccharomyces cerevisiae* is a probiotic yeast, which has beneficial effects on animal growth, host immune function, inhibition of *Salmonella* spp. adhesion or antagonist effect against *Escherichia coli* O157:H7 (Jurgens et al. 1997). Furthermore, *S. cerevisiae* has been shown to decrease inflammation in a mouse model of chemically-induced colitis (Foligne et al. 2010); however, Il-6 can be produced by *S. cerevisiae* stimulus (Seif et al. 2016), in agreement with this research. This study showed that that *S. cerevisiae* has the ability to increase IL-6 and IL-8 expressions of IPEC-J2 cells induced by DON. IL-6 and IL-8 are usually considered as pro-inflammatory cytokines (Huang et al. 2019) because they are the signals produced by the external stimuli; however, IL-6 and IL-8 are also considered as the anti-inflammatory cytokines required for controlling local or systemic acute inflammatory responses (Xing et al. 1998; Zanello et al. 2011) because both of them are the signals of inflammatory response to trigger the immune system for attacking inflammation. IL-6 and IL-8 changes may be related to nuclear factor kappa B (NF-κB) signaling (Huang et al. 2019). The further experiments revealed that *S. boulardii* effectively reversed DON-induced cytotoxicity through down-regulating the expression of TNF-α, IL-6, and IL-lβ in porcine alveolar macrophage cells (Chang et al. 2017), which does not correspond with this research, maybe due to the different responses from the different cell types and microbial species.

IL-10, known as an anti-inflammatory factor or cytokine synthesis inhibitory factor, has a variety of biological functions. It mainly inhibits activated cells to play an effective role in immune regulation. It is also an important cytokine regulator in mucosal immunity to maintain the stability of intestinal mucosal environment. This research indicated that IL-10 mRNA abundance was significantly up-regulated by *S. cerevisiae* addition only when IPEC-J2 cells were exposed to the highest DON concentration at 4 µM. It is inferred that the relieving effect of *S. cerevisiae* on IPEC-J2 cells induced by DON appears under the condition of exotic strong stimuli. The previous result indicated that *S.* *cerevisiae* was able to restore IL-10 level in the prevention of allergic diseases for mice (Fonseca et al. 2017), corresponding with this study. Another report indicated that IL-10 change may be related with NF-κB and rapamycin (TOR) signaling in juvenile grass carp (Huang et al. 2019).

Inference engine component suites are tightly packed with intercellular junction complexes that regulate paracellular permeability and integrity of the epithelial barrier (González-Mariscal et al. 2008). Some studies have indicated that DON dramatically alters barrier function and intestinal permeability via modulation of the tight junctions or mucus layer (Diesing et al. 2011a, b; Pinton et al. 2012, 2015). The previous report showed that 20 µM DON induced significant reductions of claudin-1 and claudin-3, but without influence on claudin-4, ZO-1, ZO-2, ZO-3 and occludin (Springler et al. 2016), but another research showed that 6.73 µM DON decreased occludin expressions of IPEC-J2 cells after 48 h reaction (Gu et al. 2016), in disagreement with this study. This research indicated that the expressions of TJP-1 and occludin were significantly up-regulated after IPEC-J2 cells explored to more than 0. 5–1 µM DON; however, *S. cerevisiae* addition could down-regulate TJP-1 and occludin expressions especially at 1 µM DON. It is deduced that *S. cerevisiae* is able to protect intestinal normal structure change as barrier function from damage induced by DON. The previous research indicated that DON supplementation in broiler diet significantly decreased mRNA expression of jejunal claudin-1 and occludin; however, *L. plantarum* supplementation could alleviate intestinal damage induced by DON (Wu et al. 2018). The above result is almost the same as this research because *S. cerevisiae* and *L. plantarum* are two strain probiotics to share the similar functions to detoxify mycotoxins and keep normal gut structure.

In conclusion, *S. cerevisiae* exerts a protective effect against DON-induced IPEC-J2 cell disruption by degrading DON, decreasing LDH release, alleviating cell necrosis and apoptosis, up-regulating anti-inflammatory cytokines, and down-regulating TJP genes.

## Data Availability

Not applicable.

## Abbreviations

DON: deoxynivalenol
IPEC-J2: porcine jejunal epithelia cell
LDH: lactic dehydrogenase
IL-6, IL-8 and IL-10: interleukin-6, interleukin-8, interleukin-10
TJP: tight junction proteins
CGMCC: China General Microbiological Culture Collection Center
YPD: yeast extract-peptone dextrose
CFU: colony-forming units
DMEM/F-12: Dulbecco’s modified eagle medium/nutrient mixture F-12
HEPES: 2-[4-(2-hydroxyethyl)-1-piperazinyl]ethanesulfonic acid
PBS: phosphate-buffered saline
MTT: 3-(4,5-dimethyl-2-thiazolyl)-2,5-diphenyl-2*H*-tetrazolium bromide
DMSO: dimethyl sulfoxide
OD: optical density
FITC: annexin V-fluorescein isothiocyanate
qPCR: quantitative real-time polymerase chain reaction
GAPDH: glyceraldehyde-3-phosphate dehydrogenase
SD: standard deviation
ANOVA: analysis of variance
